# Determining primary stability for adhesively stabilized dental implants

**DOI:** 10.1007/s00784-023-04990-8

**Published:** 2023-06-03

**Authors:** Ole Zoffmann Andersen, Benjamin Bellón, Maryam Lamkaouchi, Marzia Brunelli, Qiuju Wei, Philip Procter, Benjamin E. Pippenger

**Affiliations:** 1 Department of Periodontology, University of Bern, Frieburgstrasse 7, 3010 Bern, Switzerland; 2 Institut Straumann AG, Basel, Switzerland; 3 Faculty of Medicine and Health Technology, University of Tampere, Tampere, Finland; 4 University of Bordeaux, INSERM BIOTIS, U1026 Bordeaux, France; 5 Department of Materials Science and Engineering, Applied Materials Science University of Uppsala, Uppsala, Sweden

**Keywords:** Bioadhesive, Dental implant, Primary stability, Removal torque, In vitro

## Abstract

**Objectives:**

To examine factors influencing the primary stability of dental implants when stabilized in over-sized osteotomies using a calcium phosphate-based adhesive cement was the objective.

**Methods:**

Using implant removal torque measurements as a surrogate for primary stability, we examined the influence of implant design features (diameter, surface area, and thread design), along with cement gap size and curing time, on the resulting primary implant stability.

**Results:**

Removal torque values scaled with implant surface area and increasing implant diameters. Cement gap size did not alter the median removal torque values; however, larger gaps were associated with an increased spread of the measured values. Among the removal torque values measured, all were found to be above 32 Ncm which is an insertion torque threshold value commonly recommended for immediate loading protocols.

**Conclusion:**

The adhesive cement show potential for offering primary implant stability for different dental implant designs. In this study, the primary parameters influencing the measured removal torque values were the implant surface area and diameter. As the liquid cement prevents the use of insertion torque, considering the relationship between insertion and removal torque, removal torque can be considered a reliable surrogate for primary implant stability for bench and pre-clinical settings.

**Clinical relevance:**

At present, the primary stability of dental implants is linked to the quality of the host bone, the drill protocol, and the specific implant design. The adhesive cement might find applications in future clinical settings for enhancing primary stability of implants under circumstances where this cannot be achieved conventionally.

## Introduction

Dental implant stability is a critical parameter influencing the overall success of dental implant treatments [1]. The concept of dental implant stability is typically separated into two regimes, namely, I) primary stability, characterized by the purely mechanical interaction between the dental implant and the host bone and II) secondary stability, characterized by biological stabilization of the implant through the process of osseointegration [2].

Research within the field of time-dependent stability of dental implants suggests a positive correlation between primary and secondary implant stability, meaning that high primary stability is a strong indicator that the implant will reach a high secondary stability [3]. However, this is a double-edged sword as the compressive forces associated with higher insertion torque values can lead to complications such as microfractures, delayed healing, and marginal bone resorption [3, 4].

While secondary stability (osseointegration) can be determined via various methodologies, few methods exist for gauging implant primary stability. Semi-qualitative radio frequency analysis (RFA) and quantitative ultrasound (QUS) methods yield information on the dampening properties of the microenvironment in which the implant is situated, or in other words, the stiffness of the implant/bone environment [5, 6]. As a result, the readout produced by these methods does not offer a true measure of the mechanical interlocking of the implant in the bone. Contrarily, insertion torque directly produces a quantitative measure (Ncm) of the mechanical interlocking. As a result, insertion torque is widely accepted as a surrogate measure for primary stability of screw type dental implants, and it is frequently utilized for determining if an implant is eligible for immediate loading [7, 8]. But how would one determine primary stability when no mechanical interlocking with bone is present at the time of implant placement?

Using current treatment modalities, the major factors affecting primary implant stability are the surgical protocol, host bone quality and the macroscopic implant design [8]. Efforts to improve the primary stability of dental implants in poor quality bone or with reduced thread engagement are ongoing and, recently, a calcium phosphate-based cement material, having glue-like properties, has been developed [9–11]. This technology utilizes the reported properties of the amino acid phosphoserine to facilitate adhesive bonding between the cement components and, e.g., tissues and metals, under both wet and dry conditions. While this material might hold the promise to enhance the primary stability of dental implants, considering the glue-like nature and the fact that the material is a viscous liquid at the time of implant placement, insertion torque is no longer a suitable surrogate for evaluating the primary stability. Within the field of dental research, removal torque has typically been used for the assessment of secondary stability when comparing new surface technologies or implant designs in pre-clinical studies. While this method is destructive, when applied to osseointegrated implants, it comes with the benefit of a direct mechanical readout (Ncm) [12–14]. Moreover, removal torque has previously been used for assessing the stability of implants placed with the aid of an adhesive cement formulation. The study by Cochran et al. (2020) examined the use of an adhesive cement for stabilizing minimally apically engaged dental implant, *in vivo*, and used removal torque as a surrogate measure for implant stability over a healing period of up to 4 months [15]. Generally, adhesive-based primary stability generation for dental implants is not well represented in the literature and the imaginable parameters that might contribute to the stability of the initial bond have not been fully elucidated. It has been reported that the surface area of the bond interface is a key component affecting bond strength [16]. For dental implants, surface area is contingent upon 3 principal factors: implant length, implant diameter, and implant geometry (thread-to-core ratio and shape). Additionally, depending on the specific adhesive being used, setting time has been reported to play an important role in adhesive bond strength [17]. The aim of the current study was to examine factors influencing the primary stability of dental implants when stabilized in over-sized osteotomies, using a calcium phosphate-based adhesive cement. The examined factors were implant diameter, length, and thread design along with cement setting time and cement gap size. Moreover, the study focused on examining the potential for using removal torque as a surrogate measure for adhesive bond strength, thereby developing a novel indirect assessment of primary stability of dental implants placed using the adhesive cement.

## Materials and methods

### Test setup

**Fig. 1:**
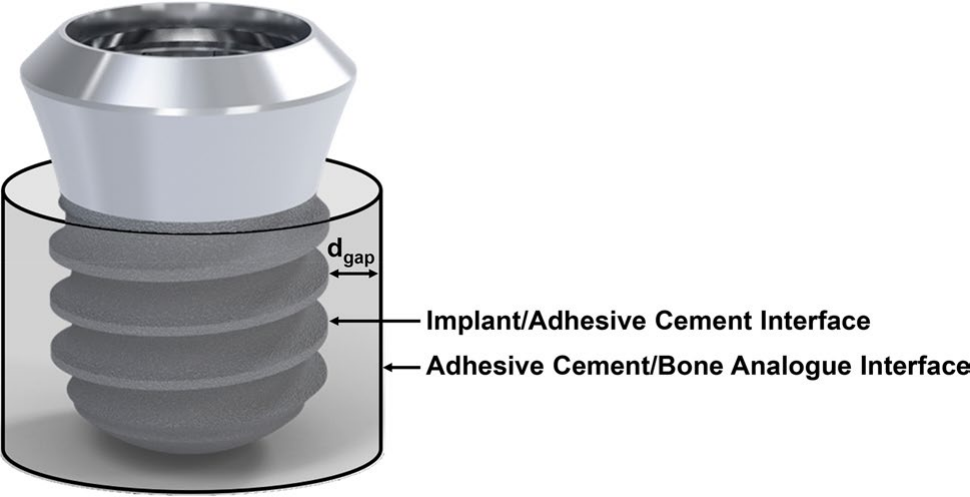
Illustration of the implant within the simulated, oversized osteotomy. The adhesive will completely fill the void between the adhesive cement/bone analog and the implant/adhesive cement interfaces. The dimension of the cement gap is determined by the distance *d*_gap_ which is measured from the wall of the simulated osteotomy to the first contact point of the implant thread

The *in vitro* bench setup utilized sawbone plates made of polyurethane (Solid Foam, 30 PCF, Sawbones Europe AB, Sweden) as a bone analogue. The bone plates were cut into rectangular blocks measuring *w*: 20 × *h*: 18 × *l*: 130 mm. Oversized cylindrical defects were then prepared in the sawbone blocks, having dimensions that would yield specific gap sizes (*d*_gap_) between the defect wall (adhesive cement/bone analogue interface) and the surface of the implant (implant/adhesive cement interface), when placed in the center of the defect, see Fig. 1. In the specific case, the term “oversized” is used to describe conditions where both the diameter and depth of the defect do not allow for the thread of the implant to engage with the sawbone material; and therefore, implant fixation is solely mediated through the presence of the adhesive cement.

**Fig. 2:**
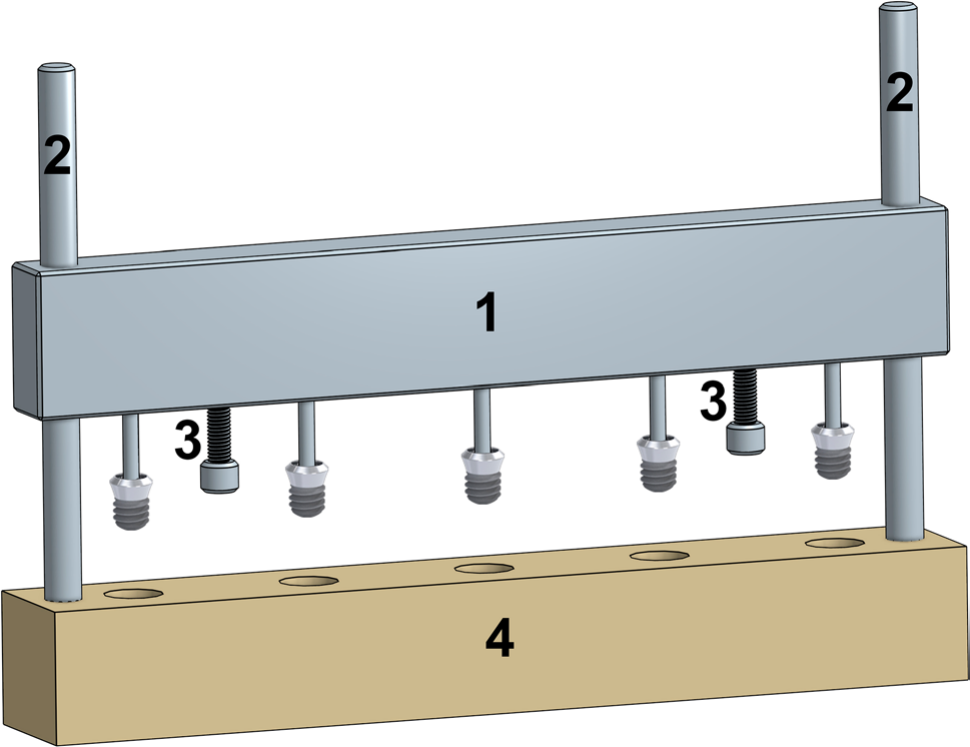
Illustration of the setup used to centrally align implants within the over-sized osteotomies. The function of the different parts are as follows: The alignment bar (1) is holding the implants during placement. The alignment bar is free to slide on the two guide rods (2), thus ensuring alignment with the osteotomies. The two screws (3) on the alignment bar allow for adjusting the depth at which the implant is placed within the simulated osteotomies prepared in the sawbone plate (4). Following placement, the jig is left in place until the time of measurement

The adhesive cement (Biomimetic Innovations Ltd., Shannon, Ireland) is stored as a dry powder and prior to implant placement 1 g of material was mixed with 250 µl of milliQ water. Mixing was performed by stirring for approximately 10 s, using a spatula. At this point, the cylindrical defects were filled with cement, followed by immediate placement of the implants. Implant placements was performed by submerging the endosteal segment of the implant into the adhesive cement, while ensuring central placement within the cylindrical defect. To ensure consistent placement of the implants, an alignment jig was utilized, see Fig. 2. Following implant placement, excess cement was removed using a spatula and the sawbone/cement/implant assembly was left to cure. Removal torque values were measured at the designated time points, with the saw bone plate being mounted in a vice to prevent rotation of the block. Torque-out values were obtained using a torque meter (MARK-10, BGI/STH50, NY, USA). To reduce the impact from variations arising from, e.g., different mixing techniques, all experiments were performed by the same operator.

### Handling and curing time

During the preliminary work, performed in preparation for this study, it was found that some level of expertise was needed to yield consistent results and that this was predominantly related to the mixing and the time window (1–2 min) within which handling of the adhesive cement was possible, without disturbing the curing process. In order to better understand the curing process, a time series was performed to determine the mechanical properties following 2, 5, 10, 15, 30, and 60 min, and 24 h, after mixing had been initiated. Additionally, a series of experiments was performed to examining the potential for extending the working time of the material by adding trisodium citrate. This was performed at the same intervals by exchanging the entire water volume with a 15% (w/v) solution of trisodium citrate (Sigma-Aldrich, Germany) in milliQ water. Both series were performed with a minimum of 8 samples per time point, using Roxolid SLActive TL Ø4.1 × 4-mm implants (Institut Straumann AG, Basel, Switzerland) and a cement gap size (*d*_gap_) of 1.5 mm.

### Effect of cement gap size

To examine the influence of the cement gap size, i.e., the distance from the surface of the implant (major diameter) to the surface of the sawbone wall, within the defect, a series of simulated osteotomies were prepared with diameters of either 5.1, 6.1, 7.1, or 10.1 mm. The Roxolid SLActive TL 4.1 × 4-mm implant (Institute Straumann AG, Basel, Switzerland) was utilized for these experiments, yielding gap sizes of either 0.5, 1.0, 1.5, or 3.0 mm. Based on the results from the curing time series, the cement was allowed to set for 15 min, prior to measuring the removal torque.

### Influence of dental implant geometry

The influence of having dental implants with varying dimensions and thread designs was investigated using the implant types presented in Table 1. To allow for examining the individual effects arising from implant length, diameter, and thread design, implants were chosen to have a specific correlation between their endosteal areas. For this part of the study, all tests were performed with a cement gap size of 1.5 mm.

**Table 1:** Overview of and the correlation between the different implant types utilized to investigate the effect of dimensions and thread design on the stability of the adhesive cement/implant interface

Implant type	Diameter (mm)	Length (mm)	Endosteal area (mm^2^)	Correlation
TL	4.1	4	66.5	Same diameter with a 1: 2 relation in surface area
TL	4.1	10	133.0	
TLX	3.75	6	86.2	Same diameter with a 1: 2 relation in surface area
TLX	3.75	12	170.1	
TLX	4.5	10	179.6	Similar surface area to TLX 3.75 × 12, but with a wider diameter

### Data analysis and statistics

Each data set comprises measurements from a minimum of 8 samples (*n* = 8). Data from single time point experiments is presented as boxplots with indication of median values and inter quartile ranges. Whiskers represent the full range of the data set, and no outliers have been excluded from the data sets. Data from the time series experiments, with and without the use of trisodium citrate, is presented as line plots with median values represented by markers. Error bars represent the full range of the data set. Independently from the type of data representation, the non-parametric Wilcoxon rank-sum test was employed to identify significant differences between data sets, as not all data sets adhered to a normal distribution. To reach a normal distribution for all data sets, a larger samples size would be appropriate for further testing.

## Results

### Handling and curing time

**Fig. 3:**
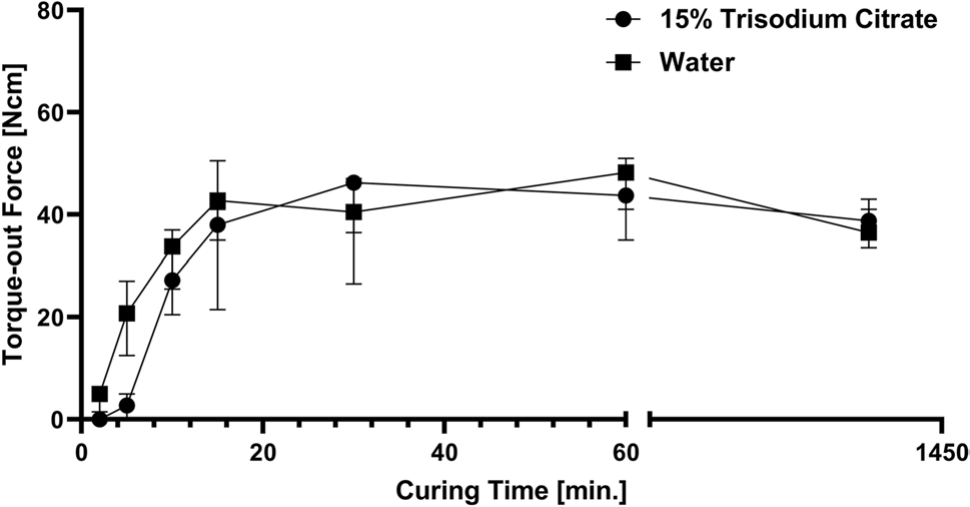
Comparison between torque-out values obtained with cement formulations comprising either water or an aqueous solution of 15% trisodium citrate. As is evident from the curve, the effect of trisodium citrate is pronounced for the early time points (at 2 and 5 min, *p* ≤ 0.01) and less pronounced at 10 and 15 min (*p* ≤ 0.05) before the two formulations reaches a similar level, with no statistical differences, for the later time points

In order to determine a single time point, at which assessment of the mechanical strength of the sawbone/adhesive cement/implant assemblies would be suitable, a time series was performed. Here, the torque-out values were determined at different time points, relative to initiating the mixing of the adhesive cement. Additionally, a series of experiments were included to investigate the potential for extending the handling time of the material, by performing the same time series with samples where the water fraction was replaced by a solution of 15% (w/v) trisodium citrate in water. The data from the time series is presented in Fig. 3. From the data, the dependence of the curing time on the mechanical strength of the material is evident. For the formulation without trisodium citrate, at 2 min, the material appears solid to the touch; however, the full strength has not been developed. The removal torque force is found to increase up to the 15 min time point at which the material has developed full strength. This is evidenced by the plateauing of the removal torque values. The use of trisodium citrate was found to extent the working time of the mixed adhesive cement, and at the 2-min time point, the material was still viscous. Significant differences are observed between the two formulations up until the 15-min time point after which the torque out values reach a comparable level.

**Fig. 4:**
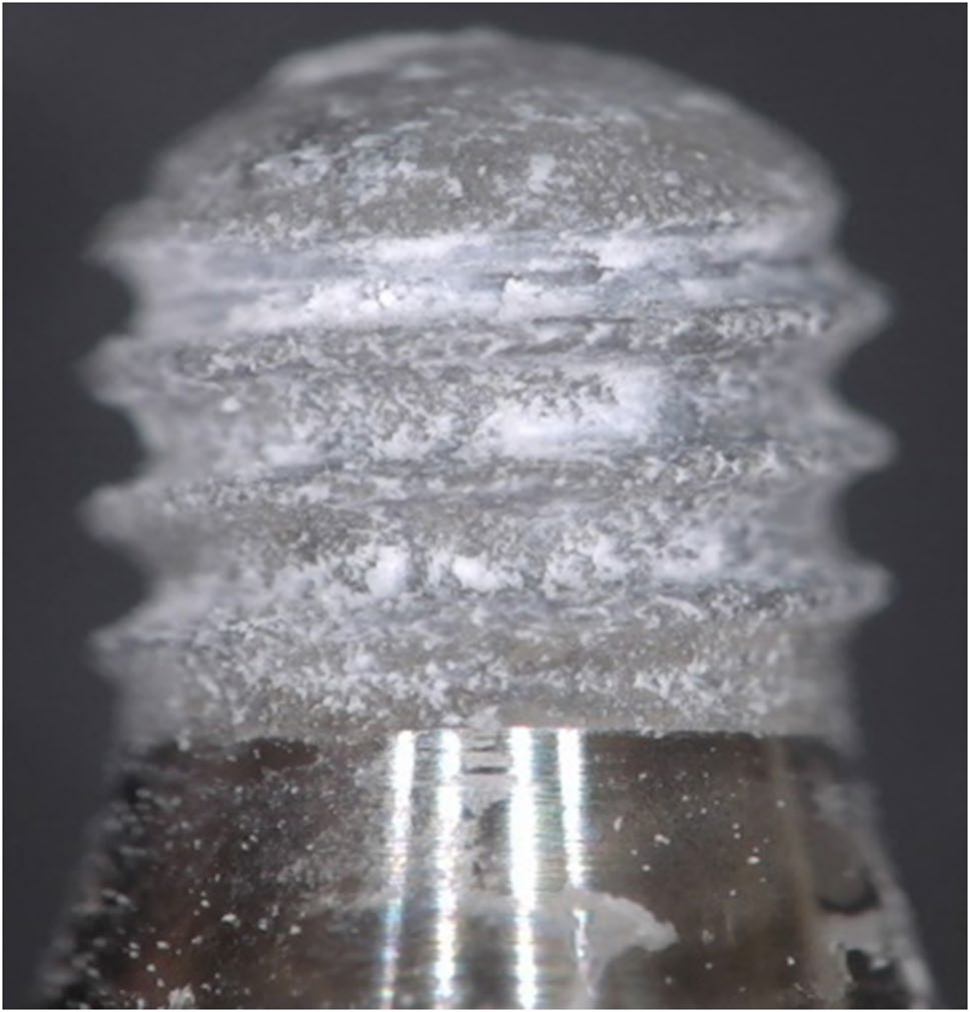
Example of light optical microscopy image of a TL Ø4.1 × 4 mm implant following torque-out from the water based adhesive cement formulation, following a curing time of 15 min. Based on the appearance of the surface, the mode of failure is predominantly adhesive in nature

Excluding the 2-min time point for the trisodium citrate formulation (material still viscous), independent of the specific formulation, failure was consistently found to occur at the adhesive cement/implant interface. Following removal, implants were found to have a dusty grey/white appearance with the presence of localized islands of adhesive cement, indicating a predominantly adhesive mode of failure, see Fig. 4.

### Cement gap size

**Fig. 5:**
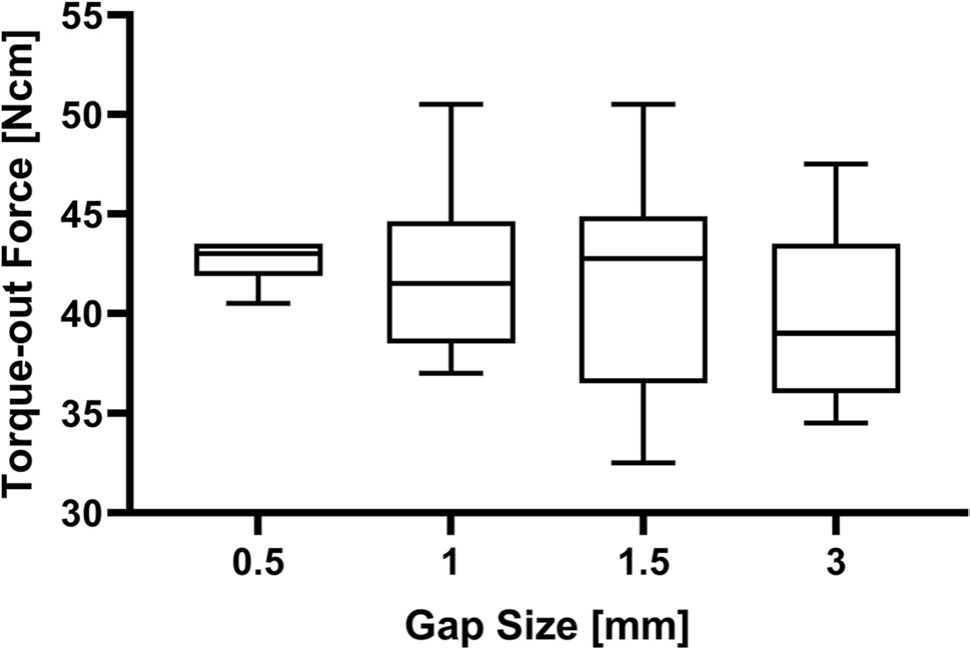
Removal torque data obtained from the samples prepared with various gap sizes using pure water, following 15 min of curing. While there is no significant difference between the groups, it appears that gap sizes ≥ 1 mm are associated with a larger spread of the data points

The correlation between gap size and removal torque was examined by preparing simulated osteotomies of varying sizes, yielding gaps of either 0.5, 1, 1.5, or 3 mm. The data presented in Fig. 5 was obtained using the formulation without trisodium citrate, after 15 min of curing. For the different gap sizes, no significant differences were found; however, for gap sizes ≥ 1 mm, a larger spread of the data is observed.

### Influence of implant geometry

**Fig. 6:**
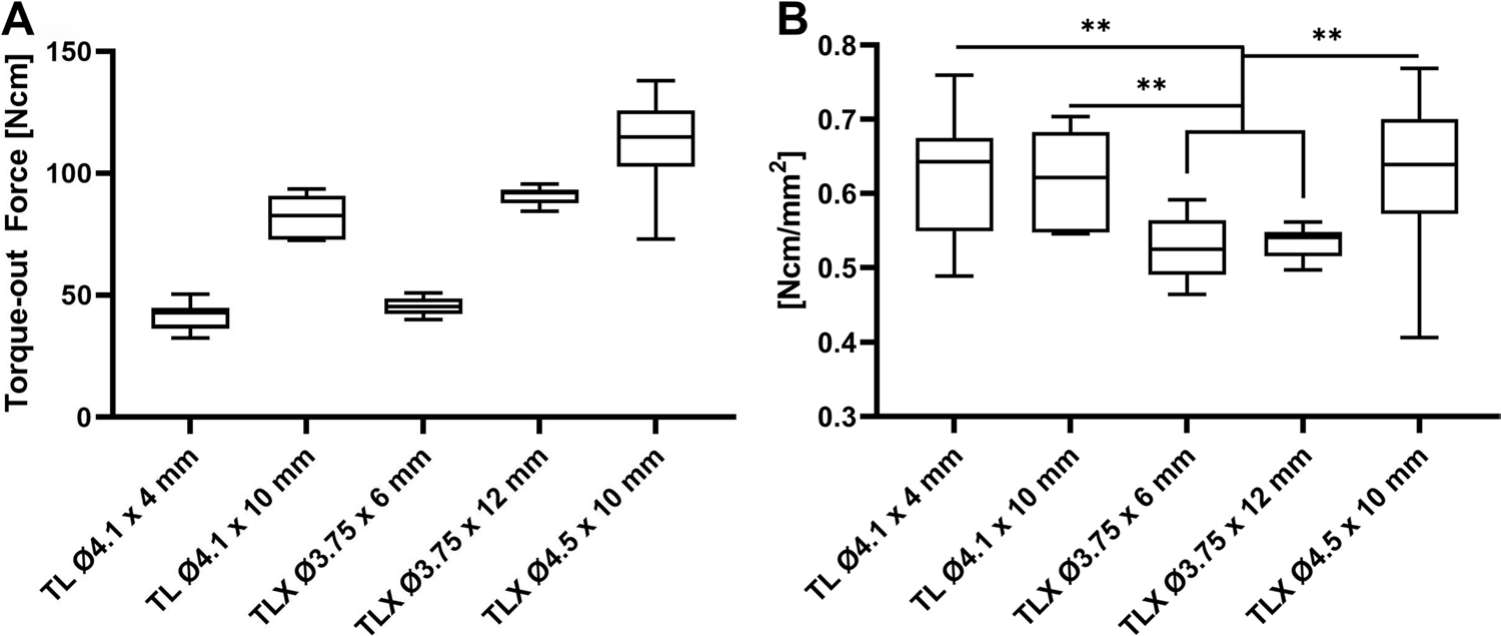
**A** Removal torque values obtained for the 5 different implant geometries. The relation between implants having the same diameter and thread design, but varying lengths, is found to be linear, as the required torque is doubled when the surface area is doubled. **B** Torque-out data normalized to the surface area of the implant. This representation shows that the implant diameter is also a contributing factor and hints towards some role of the thread design. Significant differences are found when comparing data from the other groups to the two TLX Ø3.75-mm implants. ^**^p ≤ 0.01

The data obtained from the experiments using implants with different geometrical designs is presented in Fig. 6A. Again, the experiment was performed using the pure water formulation and torque-out was performed following 15 min of curing. The predominant factors affecting the removal torque was found to be the endosteal surface area and the implant diameter. The latter is evident when comparing the three TLX implants and normalizing the measured torque values with respect to the surface area, see Fig. 6B. Considering the sequence of implant diameters (3.75, 4.1 and 4.5), the linear dependence of torque with distance/diameter and the observed significant difference between the TLX 3.75 and the TL 4.1 implant variants, one would also have expected a significant difference between TL 4.1 and TLX 4.5. This, however, is not observed and might hint towards the specific thread design having an impact on the removal torque. Generally, it appears that increasing diameter leads to higher median torque values; however, this comes at the cost of an increased spread of the data.

## Discussion

The aim of the current study was to examine factors influencing the primary stability of dental implants when stabilized in over-sized osteotomies, using a calcium phosphate-based adhesive cement. The term over-sized refers to the implant only being held in place by the cement with no engagement of the implant thread with the bone analogue material. As the adhesive cement is a viscous liquid at the point of implant placement, the standard procedure of using insertion torque to assess the primary stability of the implant is not applicable. Instead, this study utilized removal torque measurements as a surrogate measure for the primary stability, to assess if this would be a reliable approach for validating the *in vitro* performance of the adhesive cement. Moreover, the study was designed to shed light onto which parameters might influence the bonding-strength of the bone analogue material/cement/implant assembly. Prior to diving into the results, one important question remains—what would be an appropriate acceptance criterion for the strength of the bone analogue/cement/implant assembly as assessed through removal torque measurements? Considering the trend towards shorter implant treatment procedures, the ultimate goal for the adhesive cement, in clinical settings, would be to allow for immediate loading of the restoration. Using standard protocols, the current consensus is that implants placed with an insertion torque > 32 Ncm are eligible for immediate loading [18]. This is, however, still a matter of debate and several studies suggest that insertion torque values significantly below 32 Ncm does not exclude immediate loading protocols and that it might even be beneficial for the healing process [18, 19]. Independent of the specific insertion torque value, the question about the relationship between insertion and removal torque is raised—is there a direct correlation? In line with earlier findings, the study by Yamaguchi et al. (2015) studied the relationship between insertion torque and removal torque of various implant designs and placed in bone analogue materials. They found that the removal torque values are typically lower than the corresponding insertion torque value except for parallel walled implants. For this implant type, no significant difference were found between the insertion torque and removal torque values [20]. These findings highlight the relevance of using removal torque as a surrogate measure for primary implant stability, in a pre-clinical setting. While the mechanical forces associated with adhesive cement-stabilized implants are very different from conventionally stabilized implants (i.e., absence of compressive forces for the cement stabilized implants), the removal torque measurement still gauges the mechanical interlocking of the implant, within the osteotomy. As a result, it is considered relevant to benchmark the removal torque against a widely accepted insertion torque value for immediate loading protocols and, since the use of the adhesive cement does not come with the risk of peri-implant bone fractures, the worst case value of > 32 Ncm would be a relevant benchmark parameter for the further development of the adhesive cement.

Understanding the properties of the adhesive cement, and which parameters affect its behavior, is important for the further development. To start this process, a time series was prepared. This revealed that the material obtained its optimal strength after approximately 15 min. However, the handling of the material is limited to less than two minutes. Working with the material, it appeared that the ambient temperature has a major effect on the transition from liquid to solid. Additionally, it was observed that the mixing itself affects how long the material stays in its viscous state, meaning that more vigorous mixing shortens the time window in which the material can be handled. In the current study, these challenges were handled by having one skilled operator perform all experiments. However, for future studies, it would be advantageous to apply an automated mixing procedure and performed under temperature-controlled conditions. In relation to this, examining the potential for expanding the handling period with the addition of, e.g., trisodium citrate could also be an interesting route to follow further. The data from the current study indicates that it is feasible to extend this time window, and it also indicates that similar mechanical properties are obtained when the material is allowed to terminally cure. It would be interesting to expand these investigations by varying the trisodium citrate concentration, to understand the impact on the material setting time and strength.

The data obtained from the different gap sizes do not show significant differences in median values; however, it appears that larger gap sizes are accompanied by a larger spread of the data. It is known from the literature that increasing the layer thickness of an adhesive comes at the cost of reduced strength [21]. This is thought to be a result of an increased number of naturally occurring internal defects, within the adhesive layer, leading to an increased risk for a cohesive-type failure [16, 21]. From a first glance, this knowledge contradicts the findings from the current study as the mode of failure appear to be the same, independent of the gap size. Considering that the implants are torqued out, the cylindrical geometry of the implant/adhesive cement assembly and the relationship between torque and distance from the center of the implant, it is reasonable to assume that a cohesive failure is most likely to occur close to the surface of the implant. This, combined with the potential for abrasion between the implant and the cured adhesive cement during post-test retrieval of the implant, might suggest that the actual failure mode is more of a cohesive nature. To shed more light on the influence of layer thickness and failure mode, associated with the adhesive cement, it would be interesting to evaluate the removal torque in combination with, e.g., implant pull-out or standardized adhesive tensile testing.

Looking into the impact of the different geometrical factors arising from the design of the implant, it is evident that the two major contributing factors are the endosteal surface area of and the diameter of the implant. Additionally, the data suggests that the specific implant thread design might influence the primary stability of the implant. Here, the pronounced threads of the TLX implant design appear to have a negative impact on the measured removal torque. This is hypothesized to be a combined effect of the relatively deep and narrow space between the threads, compared to the threads of the TL implant, and relatively high viscosity of the adhesive cement mixture, potentially preventing optimal contact between the adhesive and the full extended of the implant surface. For future studies, it would be interesting to further assess the impact of thread design and, moreover, assess the impact of parameters such as tapering and self-cutting geometries.

The translation of the findings from the current bench study into an *in vivo* setting remains to be confirmed and, in particular, the failure mode as healing progresses. Would there, e.g., be some form of stability dip when an implant is primarily stabilized by the adhesive cement? Additionally, hypothesizing that the conversion of the adhesive cement to bone would begin at the adhesive cement/bone interface, more narrow gap sizes would lead to new bone at the implant surface earlier and this may be detectable through assessment of the implant removal torque at different time points.

## Conclusion

For the tested dental implants, removal torque values were found to increase with increasing surface area and an increasing implant diameter was also associated with increasing torque values. The specific thread design of the implant appear to impact the resulting removal torque values. This, however, must be confirmed through testing a wider range of implants. Considering the removal torque values obtained for this study and the current consensus that insertion torque values of 32 Ncm are suitable for immediate loading protocols, the further development of this material for clinical applications may hold the potential for reducing the number of surgical interventions, associated with current treatment protocols. As the viscous nature of the pre-set cement prevents the use of insertion torque, taking the general relationship between insertion and removal torque into account, removal torque is considered a reliable surrogate measure for primary implant stability for bench and pre-clinical settings.

## Data Availability

The data making the foundation for this research is available upon request, by contacting the corresponding author Benjamin E. Pippenger, University of Bern, Department of Periodontology, Frieburgstrasse 7, 3010 Bern, Switzerland (email: benjamin.pippenger@unibe.ch, tel: + 41 31 632 25 77).

## References

[1] Javed F (2013). Role of primary stability for successful osseointegration of dental implants: factors of influence and evaluation. Interv Med Appl Sci IMAS.

[2] Meredith N (1998). Assessment of implant stability as a prognostic determinant. Int J Prosthodont.

[3] Monje A (2019). Relationship between primary/mechanical and secondary/biological implant stability. Int J Oral Maxillofac Implants.

[4] Marconcini S (2018). Longitudinal analysis on the effect of insertion torque on delayed single implants: a 3-year randomized clinical study. Clin Implant Dent Relat Res.

[5] Hériveaux Y (2021). Assessment of dental implant stability using resonance frequency analysis and quantitative ultrasound methods. J Prosthodont Res.

[6] Bafijari D (2019). Influence of resonance frequency analysis (RFA) measurements for successful osseointegration of dental implants during the healing period and its impact on implant assessed by Osstell mentor device. Open Access Maced J Med Sci.

[7] Giudice R (2019). Implant insertion torque value in immediate loading: a retrospective study. Medicina Oral Patología Oral y Cirugia Bucal.

[8] Tettamanti L (2017). Immediate loading implants: review of the critical aspects. Oral Implantol (Rome).

[9] Bystrom JL, Pujari-Palmer M (2019) Phosphoserine functionalized cements preserve metastable phases, and reprecipitate octacalcium phosphate, hydroxyapatite, dicalcium phosphate, and amorphous calcium phosphate, during degradation, in vitro. J Funct Biomater 10(4):54 10.3390/jfb10040054 PMC6963472 31783637

[10] Pujari-Palmer M et al (2018) A novel class of injectable bioceramics that glue tissues and biomaterials. Materials (Basel) 11(12):2492 10.3390/ma11122492 PMC6316977 30544596

[11] Pujari-Palmer M (2020). Factors that determine the adhesive strength in a bioinspired bone tissue adhesive. ChemEngineering.

[12] Park EY, Sohn HO, Kim EK (2019). Comparison of the removal torque and a histomorphometric evaluation of the RBM treated implants with the RBM followed by laser treated implants: an experimental study in rabbits. Yeungnam Univ J Med.

[13] Silva-Boghossian C et al (2017) Removal torque and bone adherence to dental implants surface. J Dent Health Oral Disord Therapy 8(2)

[14] Cho S-A, Park K-T (2003). The removal torque of titanium screw inserted in rabbit tibia treated by dual acid etching. Biomaterials.

[15] Cochran DL (2020). Immediate dental implant stabilization in a canine model using a novel mineral-organic adhesive: 4-month results. Int J Oral Maxillofac Implants.

[16] von Fraunhofer JA (2012). Adhesion and cohesion. Int J Dent.

[17] Odabaş ME, Bani M, Tirali RE (2013). Shear bond strengths of different adhesive systems to biodentine. ScientificWorldJournal.

[18] Del Giudice R (2019). Implant insertion torque value in immediate loading: a retrospective study. Med Oral Patol Oral Cir Bucal.

[19] Norton MR (2017). The influence of low insertion torque on primary stability, implant survival, and maintenance of marginal bone levels: a closed-cohort prospective study. Int J Oral Maxillofac Implants.

[20] Yamaguchi Y (2015). Effect of implant design on primary stability using torque-time curves in artificial bone. Int J Implant Dent.

[21] Rośkowicz M et al (2021) The effect of adhesive layer thickness on joint static strength. Materials (Basel) 14(6):1499 10.3390/ma14061499 PMC8003201 33803848

